# TRPC5-induced autophagy promotes drug resistance in breast carcinoma *via* CaMKKβ/AMPKα/mTOR pathway

**DOI:** 10.1038/s41598-017-03230-w

**Published:** 2017-06-09

**Authors:** Peng Zhang, Xiaoyu Liu, Hongjuan Li, Zhen Chen, Xiaoqiang Yao, Jian Jin, Xin Ma

**Affiliations:** 1 Wuxi School of Medicine, Jiangnan University, Wuxi, China; 2 School of Pharmaceutical Sciences, Jiangnan University, Wuxi, China; 3 School of Biomedical Sciences, The Chinese University of Hong Kong, Shatin, New Territories, Hong Kong China

## Abstract

Adriamycin is a first-line chemotherapy agent against cancer, but the development of resistance has become a major problem. Although autophagy is considered to be an adaptive survival response in response to chemotherapy and may be associated with chemoresistance, its inducer and the underlying molecular mechanisms remain unclear. Here, we demonstrate that adriamycin up-regulates the both levels of TRPC5 and autophagy, and the increase in autophagy is mediated by TRPC5 in breast cancer cells. Blockade of TRPC5 or autophagy increased the sensitivity to chemotherapy *in vitro* and *in vivo*. Notably, we revealed a positive correlation between TRPC5 and the autophagy-associated protein LC3 in paired patients with or without anthracycline-taxane-based chemotherapy. Furthermore, pharmacological inhibition and gene-silencing showed that the cytoprotective autophagy mediated by TRPC5 during adriamycin treatment is dependent on the CaMKKβ/AMPKα/mTOR pathway. Moreover, adriamycin-resistant MCF-7/ADM cells maintained a high basal level of autophagy, and silencing of TRPC5 and inhibition of autophagy counteracted the resistance to adriamycin. Thus, our results revealed a novel role of TRPC5 as an inducer of autophagy, and this suggests a novel mechanism of drug resistance in chemotherapy for breast cancer.

## Introduction

Breast cancer is the most common malignancy and the major cause of cancer-related death among women worldwide^1, 2^, while adriamycin (ADM), is the first-line chemotherapy agent against this form of cancer. However, the acquisition of ADM resistance is a leading cause of treatment failure^3–5^. It has been demonstrated that impairment of apoptotic signaling is the main mechanism of drug resistance^6–8^, but the underlying mechanisms are still not entirely clear. Therefore, understanding the signaling pathways underling the resistance of breast cancer cells to chemotherapy is critical for the development of novel therapeutic strategies.

Human canonical transient receptor potential channel 5 (TRPC5) is a Ca^2+^-permeable cation channel that is expressed in many types of cells^9–11^, and is involved in several neuronal and vascular diseases^12–14^. Our previous studies have demonstrated that TRPC5 is associated with cancer chemotherapy. Our findings showed that overexpression of TRPC5 induces chemoresistance by up-regulating of p-glycoprotein and hypoxia-inducible factor-1α in chemoresistant breast cancer cells^15, 16^. Our recent studies have suggested that the level of TRPC5 in circulating extracellular vesicles may be correlated with the clinical response to chemotherapy^17^. Therefore, TRPC5 may be a good molecular target for the diagnosis and treatment of breast cancer.

Macroautophagy (here referred to simply as autophagy), an evolutionarily-conserved lysosomal pathway, is functions in the degradation of long-lived proteins, cellular macromolecules, and whole organelles^18, 19^. It is characterized by the formation of a closed double-membrane vacuole, named the autophagosome, which matures in a stepwise process involving engulfing events and fusion with endolysosomal vesicles^20, 21^. Lysosomal hydrolases digest the contents of autolysosomes to recyclable breakdown products, generating energy to confer stress tolerance^22, 23^. Autophagy also plays a critical role in cancer development and death^24–26^. While some reports have indicated that this process can support cancer cell survival^27–29^, in contrast, several studies have suggested that autophagy promotes cancer cell death^30, 31^. The different roles of autophagy in cancer seem to depend on tumor type, stage, genomic context and setting^26^. In general, autophagy suppresses tumor initiation, but it promotes tumor progression and is considered to be a key survival pathway in response to stress. A number of anticancer drugs induce the apoptotic cell death pathway while simultaneously triggering an autophagic response. In cancer therapy, most of the data point to autophagy as a protective pathway that delays apoptotic cell death. The inhibition of autophagy significantly enhances the cell death induced by epirubicin in MCF-7 cells and triple-negative breast cancer cells^32, 33^. However, the mechanism by which chemotherapy induces this protective autophagy and the identity of the inducing factor remain unclear.

Given these findings, we hypothesized that TRPC5 is the inducer that initiates autophagy during chemotherapy. Therefore, we set out to test this hypothesis by investigating the relationship between TRPC5 and autophagy in drug sensitive/resistance breast cancer cells. We found that TRPC5-regulated autophagy contributes to development of chemotherapy resistance in drug sensitive breast cancer cells and maintenance of drug resistance in MCF-7/ADM cells, which is linked to Ca^2+^/calmodulin-dependent protein kinase kinase β (CaMKKβ)/ AMP-activated protein kinaseα (AMPKα)/ mammalian target of rapamycin (mTOR) signaling pathway. These results revealed a novel role of TRPC5 as an inducer of autophagy, which may suggest a novel mechanism of drug resistance in chemotherapy for breast cancer.

## Results

### Chemotherapy increases TRPC5 expression and autophagy in breast carcinoma cells

**Figure 1:**
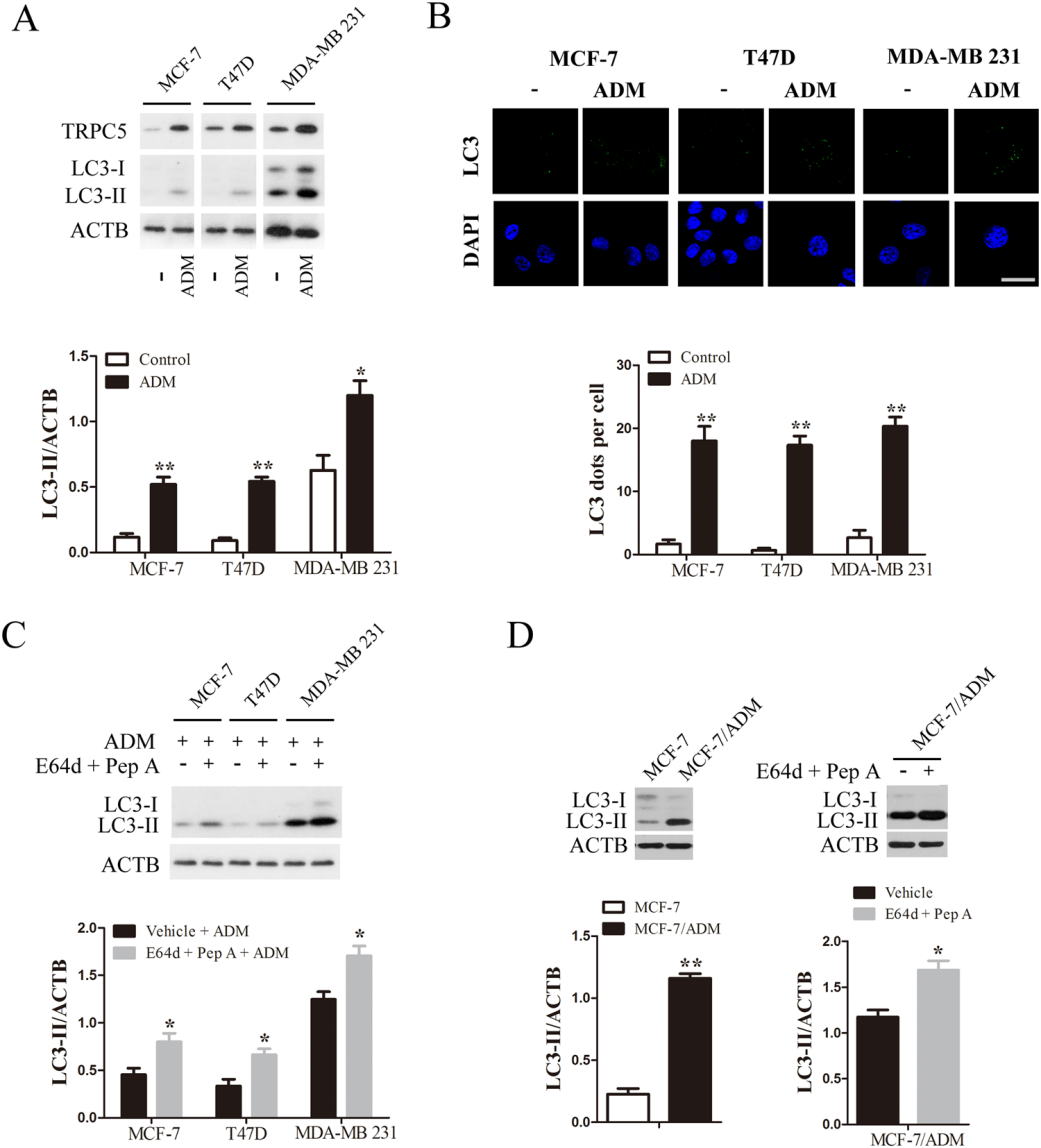
Chemotherapy enhances autophagy and TRPC5 expression in breast carcinoma cells. (**A**) Breast cancer cells were treated with 400, 300 and 800 nmol/L ADM for 48 h, and then the expression of TRPC5, LC3 and ACTB/β-actin was analyzed by western blot. Representative western blot and densitometric analysis normalized to ACTB demonstrating the effect of ADM on LC3-II levels. (**B**) Representative immunofluorescence images showing redistribution of the autophagic marker LC3 in breast cancer cells were captured on a confocal microscope, and the average number of LC3 dots per cell. Scale bar: 20 μm. (**C**) Representative western blots and densitometric analysis normalized to ACTB demonstrating the effect of the lysosomal protease inhibitors 10 μg/mL E64d plus pepstatin A (Pep A) on ADM-induced LC3-II accumulation. (**D**) Representative western blots and densitometric analysis normalized to ACTB demonstrating the LC3-II levels in MCF-7/ADM cells and the effect of E64d and Pep A. Values are means ± SEM of 4 to 6 experiments. *p < 0.05, **p < 0.01, compared to control or vehicle.

To determine whether chemotherapy enhances TRPC5 expression and autophagy in breast carcinoma cells, we detected microtubule-associated protein 1 light chain 3 (LC3-I and LC3-II) and TRPC5 by western blotting. LC3-II is a reliable marker of autophagy, specifically associated with the development and maturation of autophagosomes. MCF-7, T47D, and MDA-MB 231 cells were exposed to 400, 300, and 800nmol/L ADM respectively for 48 h after which their viability on exposure to ADM was reduced, as assessed by MTT (Supplementary Figure S1). Also the ADM exposure markedly increased their TRPC5 and LC3-II levels (Fig. 1A). Transcriptional alterations of TRPC5 were also assayed by quantitative real-time PCR and the results revealed that TRPC5 mRNA expression was increased after treatment with ADM (Supplementary Figure S2A). The increased expression of TRPC5 was time dependent in the case of MCF-7 and MDA-MB 231 cells during ADM treatment (Supplementary Figure S2B). In addition, LC3 puncta formation was tested by fluorescent imaging analysis. The number of LC3 dots per cell was significantly higher in cells exposed to ADM than in those without drug exposure (Fig. 1B). Next, we assessed the autophagic flux in cells exposure to ADM with or without the lysosomal protease inhibitors E64d and pepstatin A, which have been used to distinguish between the induction of autophagy and the suppression of autophagic vesicle degradation. We found that E64d and pepstatinA could further increased the LC3-II levels, suggesting that the increased LC3-II levels were attributable to promotion of autophagy but not to disruption of autophagic degradation (Fig. 1C) (Supplementary Figure S3). To further confirm our results, we explored autophagy in ADM-resistant human breast cancer cells (MCF-7/ADM), generated by stepwise increasing concentrations of ADM over 8 months. As shown in Fig. 1D, drug-resistant MCF-7/ADM cells had higher LC3-II expression than MCF-7 cells. Lysosomal protease inhibitors further increased the LC3-II level in MCF-7/ADM cells (Fig. 1D). These experiments showed that MCF-7/ADM cells have enhanced basal levels of autophagy. Taken together, our results suggested that TRPC5 and autophagy are both up-regulated breast carcinoma cells during ADM exposure.

### Chemotherapy-induced autophagy is regulated by TRPC5 in breast carcinoma cells

**Figure 2:**
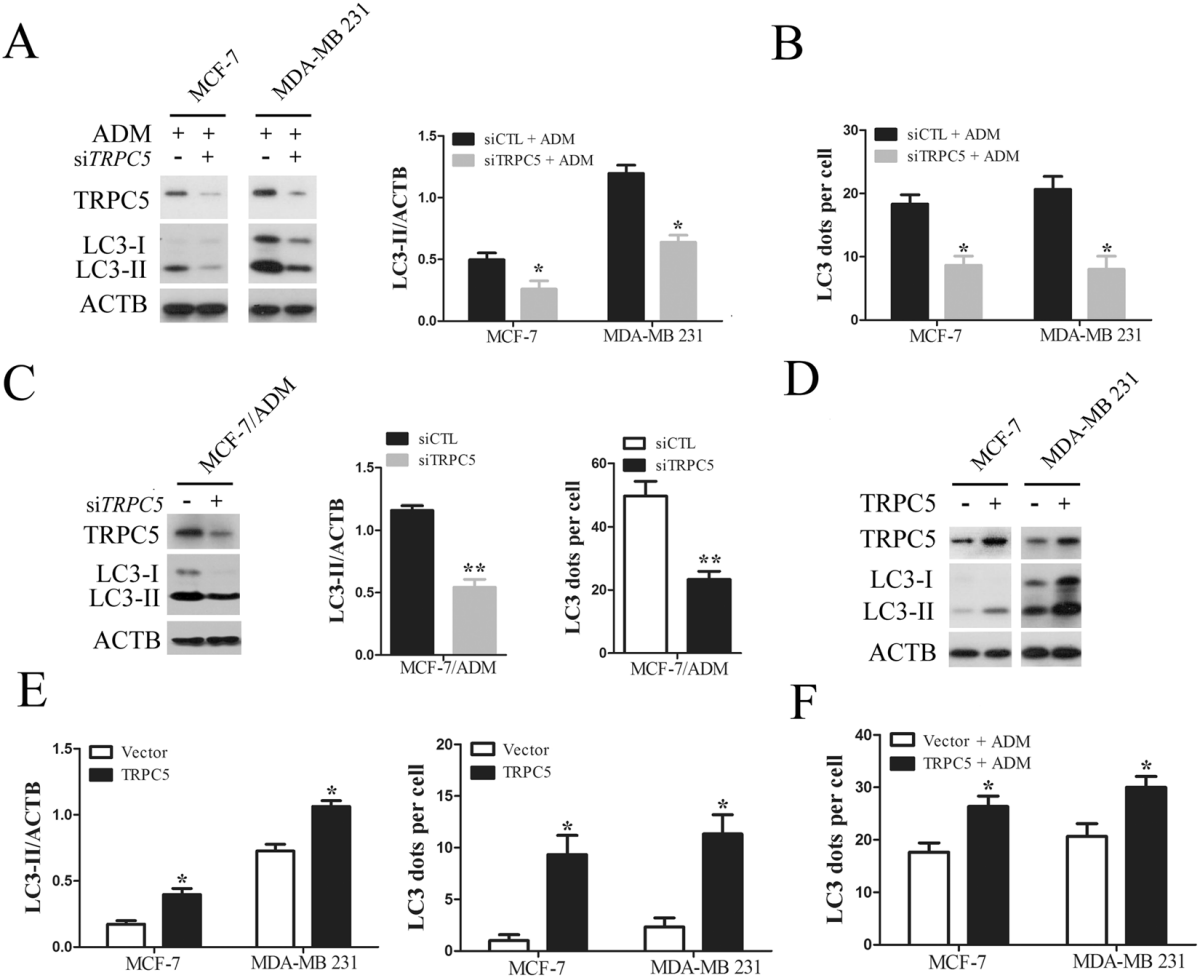
Chemotherapy induced autophagy is regulated by TRPC5 in breast carcinoma cells. (**A**) MCF-7 and MDA-MB 231 cells were transfected with siTRPC5 or siCTL for 24 h and then exposed to ADM for 48 h. The levels of TRPC5, LC3, and ACTB were quantified by Western blot. (**B**) The effect of TRPC5 knockdown on the average number of LC3 dots per cell in the indicated cells. (**C**) Representative western blots and densitometric analysis normalized to ACTB demonstrating the effect of TRPC5 silencing on the accumulation of LC3-II and the average number of LC3 dots per cell in MCF-7/ADM cells. (**D** and **E**) Representative western blot and densitometric analysis normalized to ACTB demonstrating the effect of TRPC5 overexpression on accumulation of LC3-II and average number of LC3 dots per cell in indicated cells. (**F**) The effect of TRPC5 overexpression on ADM-induced LC3 puncta formation in indicated cells. Values are mean ± SEM of 3 to 6 experiments. *p < 0.05, **p < 0.01, compared to siCTL or vector.

To determine whether TRPC5 regulates autophagy in response to ADM, we assessed the LC3-II level and LC3 puncta formation in breast cancer cells transfected with or without TRPC5 siRNA. Knockdown of TRPC5 markedly decreased the amount of TRPC5 and the LC3-II level in breast carcinoma cells exposed to ADM (Fig. 2A). The number of LC3 dots per cell was significantly attenuated after TRPC5-silencing and ADM exposure (Fig. 2B). Moreover, TRPC5 knockdown decreased the LC3-II level and LC3 puncta formation in drug-resistant MCF-7/ADM cells (Fig. 2C). To further investigate the role of TRPC5-induced autophagy in breast cancer cells during chemotherapy, we transfected MCF-7 and MDA-MB 231 cells with full-length human TRPC5 and found that overexpression of TRPC5 significantly increased LC3-II levels and LC3 puncta formation (Fig. 2D and E). LC3 puncta formation was also significantly enhanced by TRPC5 overexpression in breast cancer cells under chemotherapy (Fig. 2F). Additionally, TRPC5-silencing or -overexpression did not change the LC3 mRNA level (Supplementary Figure S4). Together, our data suggested that chemotherapy-induced autophagy is regulated by TRPC5 in breast carcinoma cells.

### Silencing of TRPC5 or inhibition of autophagy increases the sensitivity of breast carcinoma cells to chemotherapy

**Figure 3:**
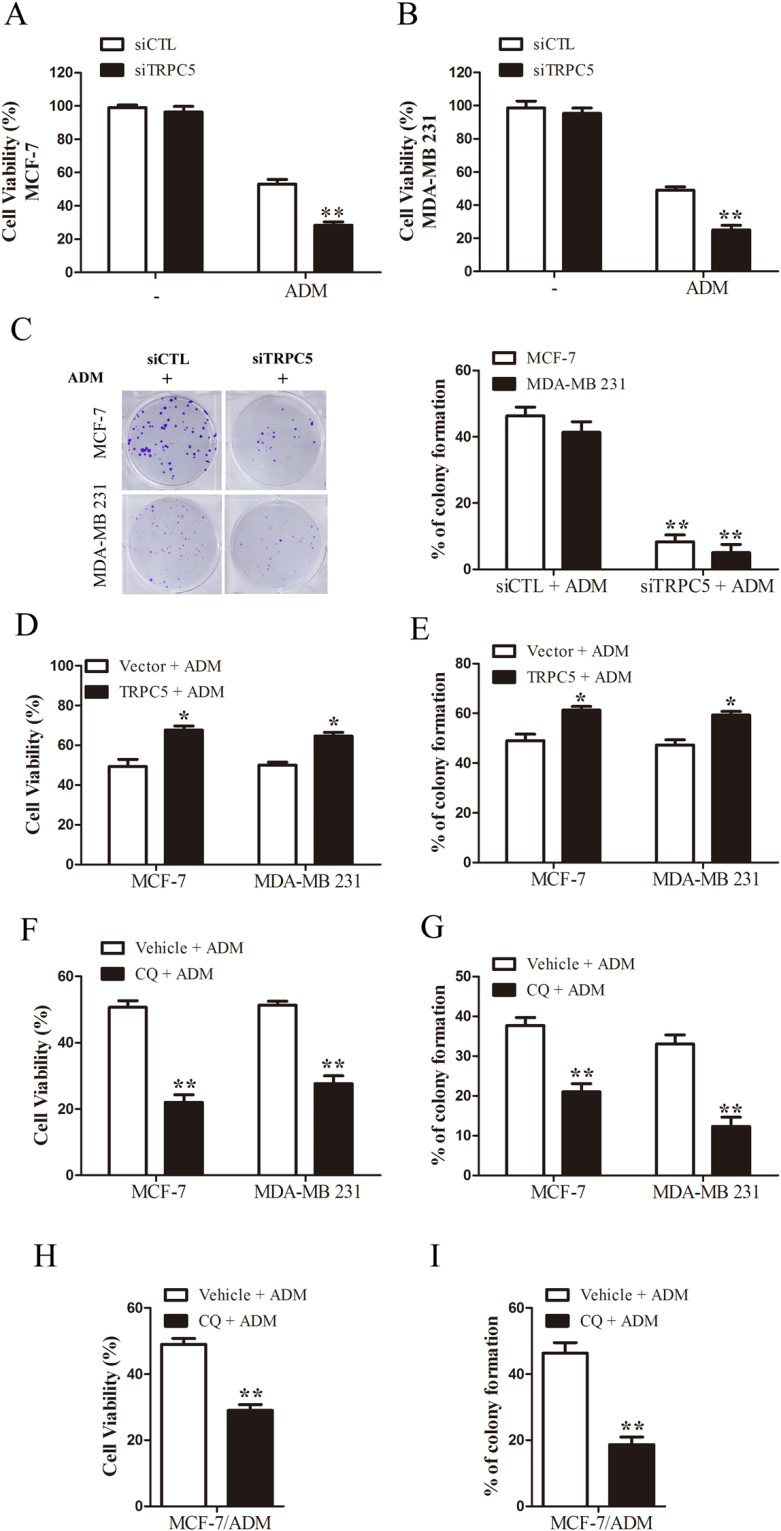
Silencing of TRPC5 or inhibition of autophagy increases the sensitivity of breast carcinoma cells to chemotherapy (**A** and **B**) MTT assays. MCF-7 and MDA-MB 231 cells were transfected with siTRPC5 or siCTL for 24 h and then exposed to 400 and 800 nmol/L ADM respectively, for 48 h. (**C**) Clonogenic recovery assays. MCF-7 and MDA-MB 231 cells transfected with siTRPC5 or siCTL for 24 h and then exposed to 5 and 10 nmol/L ADM respectively. (**D** and **E**) The effect of TRPC5 overexpression on cell viability and clonogenic recovery in indicated cells during ADM exposure. (**F**) The effect of 50 µmol/L chloroquine (CQ) on cell viability. (**G**) The effect of 3 µmol/L CQ on clonogenic recovery in indicated cells. (**H** and **I**) The effect of 50 µmol/L CQ on viability and 3 µmol/L CQ on clonogenic recovery in MCF-7/ADM cells. Values are mean ± SEM of 4 to 6 experiments. *p < 0.05, **p < 0.01, compared to siCTL, vehicle or vector.

To determine whether TRPC5-induced autophagy plays a role in the regulation of cell death in response to ADM, siRNA against TRPC5 was transfected into MCF-7 and MDA-MB 231cells. Knockdown of TRPC5 expression made these cells more sensitive to ADM-induced damage (Fig. 3A and B). We also investigate the recovery and proliferation of these cells using clonogenic crystal violet recovery assays. The results showed that 43% of MCF-7 cells proliferated cells in 5 nmol/L ADM and 38% of MDA-MB 231 cells proliferated in 10 nmol/L ADM relative to untreated cells (Supplementary Figure S5A). Knockdown of TRPC5 was more effective in reducing proliferation with 8% of MCF-7 cells and 5% of MDA-MB 231 cells remaining under ADM exposure relative to siRNA control (Fig. 3C). Moreover, we also determined the effect of TRPC5 suppression in the drug-resistant MCF-7/ADM cells, which showed a significant reduction in proliferation compared with ADM exposure alone (Supplementary Figure S5B and C). TRPC5-overexpressing cells became more resistant to ADM-induced injury and this was associated with greater recovery of cells under ADM exposure (Fig. 3D and E). In addition, chloroquine (CQ, an inhibitor of lysosomal acidification, used as a pharmacological inhibitor of autophagy) or 3-Methyladenine (3-MA) resulted in reduced viability and less colony formation with ADM in MCF-7 and MDA-MB 231 cells (Fig. 3F and G) (Supplementary Figure S6A and B). CQ and 3-MA also decreased the recovery and proliferation of drug-resistant MCF-7/ADM cells relative to ADM alone (Fig. 3H and I) (Supplementary Figure S6C and D). These results suggested that silencing of TRPC5 or inhibition of autophagy increases the sensitivity of breast carcinoma cells to chemotherapy.

### TRPC5 induces autophagy *via* the CaMKKβ/AMPKα/mTOR pathway in response to chemotherapy

**Figure 4:**
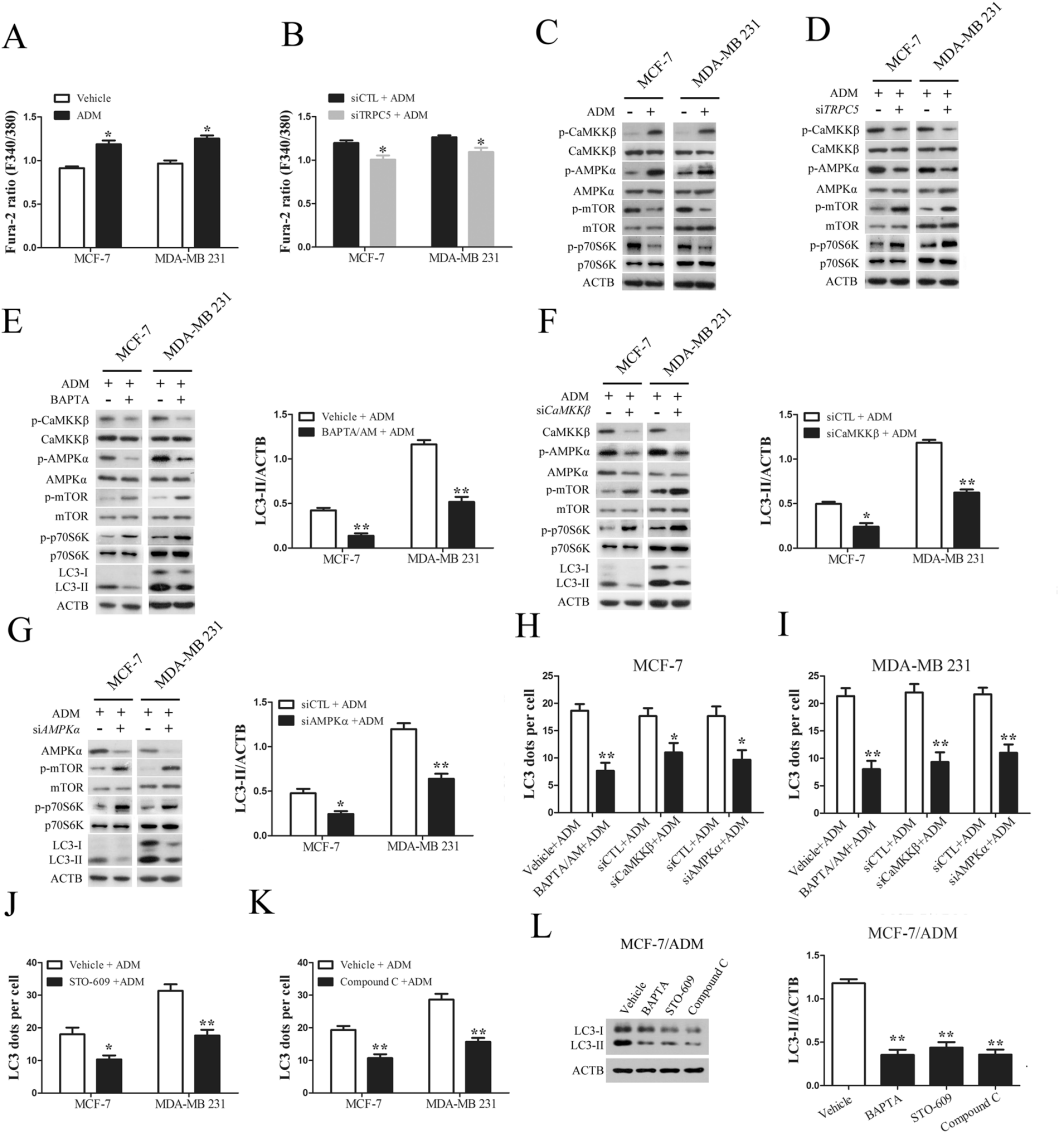
TRPC5 induces autophagy *via* the CaMKKβ/AMPKα/mTOR pathway in response to chemotherapy. (**A** and **B**) Treatment of indicated cells with ADM for 48 h increased the basal [Ca^2+^]_i_ and siTRPC5 blocked the ADM-induced increase in [Ca^2+^]_i_. (**C**) Exposure of indicated cells to ADM for 48 h was followed by analysis of the protein levels of p-CaMKKβ, CaMKKβ, p-AMPKα, AMPKα, p-mTOR, mTOR, p-p70S6K, p70S6K and ACTB by western blot. (**D**) The effect of TRPC5 silencing on the protein levels of p-CaMKKβ, CaMKKβ, p-AMPKα, AMPKα, p-mTOR, mTOR, p-p70S6K, p70S6K and ACTB in indicated cells exposed to ADM. (**E**) The effect of 20 µmol/L BAPTA/AM on the protein levels of p-CaMKKβ, CaMKKβ, p-AMPKα, AMPKα, p-mTOR, mTOR, p-p70S6K, p70S6K, LC3 and ACTB in indicated cells exposed to ADM. (**F**) The effect of CaMKKβ silencing on the protein of CaMKKβ, p-AMPKα, AMPKα, p-mTOR, mTOR, p-p70S6K, p70S6K, LC3 and ACTB in indicated cells treated with ADM. (**G**) The effect of AMPKα silencing on the protein levels of AMPKα, p-mTOR, mTOR, p-p70S6K, p70S6K, LC3 and ACTB in indicated cells exposed to ADM. (**H** and **I**) The effect of BAPTA/AM, siCaMKKβ and siAMPKα on ADM induced LC3 puncta formation in indicated cells. (**J** and **K**) The effect of 10 µmol/L STO-609 and 5 µmol/L compound C on ADM induced LC3 puncta formation in indicated cells. (**L**) The effect of BAPTA/AM, STO-609 and compound C on accumulation of LC3-II in MCF-7/ADM cells. Values are mean ± SEM of 3 to 6 experiments. *p < 0.05, **p < 0.01, compared to siCTL or vehicle.

**Figure 5:**
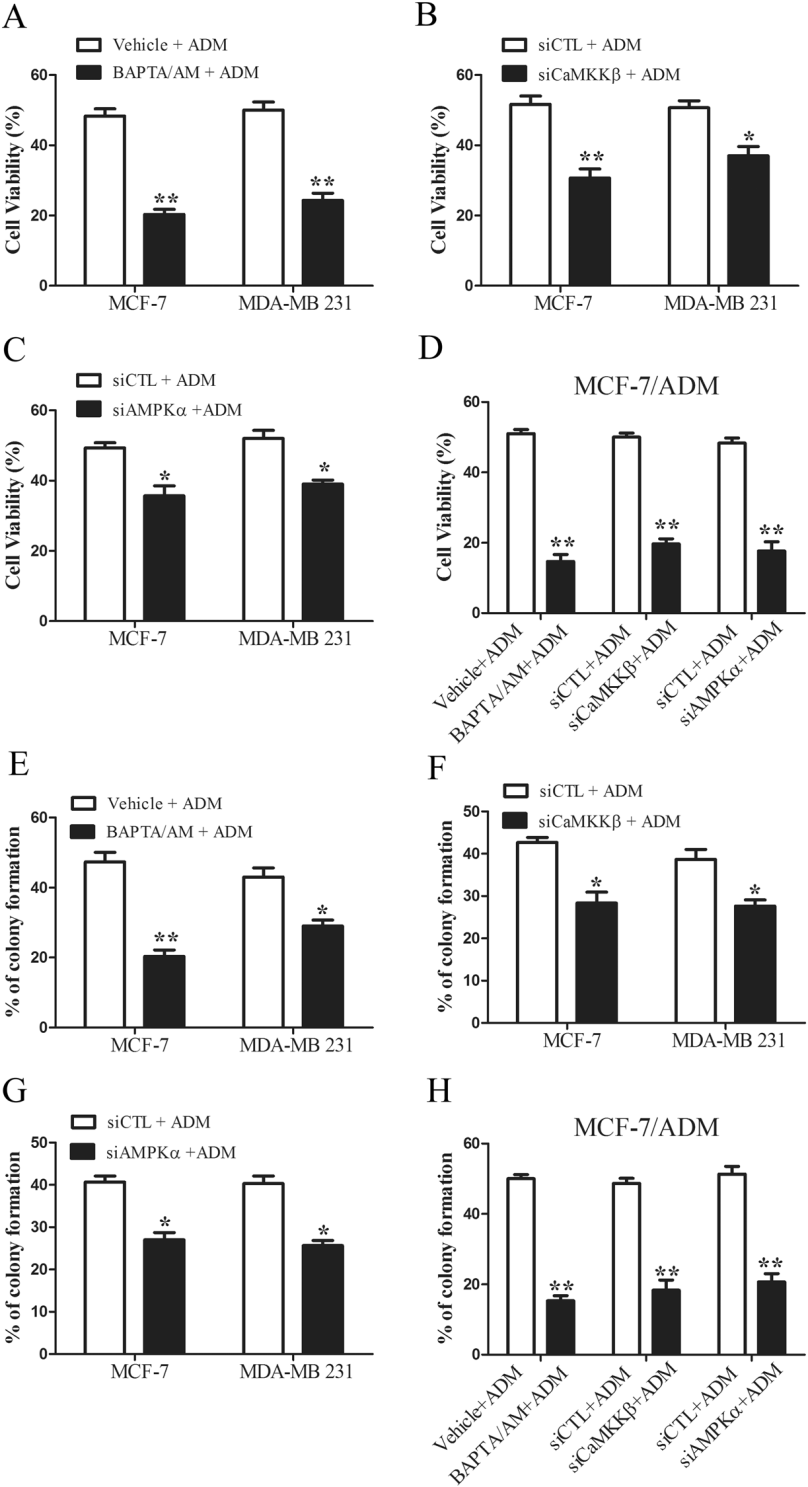
Inhibition of the CaMKKβ/AMPKα/mTOR pathway increases the sensitivity of breast carcinoma cells to chemotherapy. (**A**–**C**) The effect of BAPTA/AM, siCaMKKβ and siAMPKα on ADM-induced injury in breast cancer cells. Cell viability was assessed by MTT assays. (**D**) The effect of BAPTA/AM, siCaMKKβ and siAMPKα on ADM-induced cell injury in MCF-7/ADM cells. Cell viability was assessed by the MTT assays. (**E**–**G**) The effect of BAPTA/AM, siCaMKKβ and siAMPKα on ADM-induced colony formation in breast cancer cells. (**H**) The effect of BAPTA/AM, siCaMKKβ and siAMPKα on ADM-induced colony formation in MCF-7/ADM cells. Values are mean ± SEM of 3 to 5 experiments. *p < 0.05, **p < 0.01, compared to siCTL or vehicle.

To address the potential mechanism by which TRPC5 initiates autophagy, we investigated the downstream of TRPC5 and autophagy-associated kinases. As shown in Fig. 4A and B, we found that ADM exposure increased the cytosolic Ca^2+^ concentration ([Ca^2+^]_i_) and knockdown of TRPC5 decreased the [Ca^2+^]_i_ in MCF-7 and MDA-MB 231 cells (Supplementary Figure S7). Overexpression of TRPC5 increased the [Ca^2+^]_i_ in MCF-7 and MDA-MB 231 cells (Supplementary Figure S8). After exposing MCF-7 and MDA-MB 231 cells to ADM, we found significant increases of the phosphorylated CaMKKβ level, which occurs downstream of TRPC5 (Fig. 4C)^34^. Previous studies have shown that CaMKKβ activates AMPKα by phosphorylation^35, 36^. Consistent with this finding, AMPKα activity was markedly increased in both cell lines under ADM exposure (Fig. 4C). It has been reported that AMPKα activates autophagy by down-regulating the activity of mTOR^37, 38^. Our results showed that the level of phosphorylated mTOR and p70S6K was greatly decreased after exposure to ADM (Fig. 4C). These findings suggested that the CaMKKβ/AMPKα/mTOR pathway may be required for TRPC5 induced autophagy in response to ADM. In order to further explore whether ADM exposure leads to enhanced TRPC5 expression, which would allow a higher [Ca^2+^]_i_ to active CaMKKβ/AMPKα/mTOR-mediated autophagy, we silenced the TRPC5 expression with siRNA and assessed the activity of this pathway. We found that suppressing of TRPC5 expression significantly decreased the levels of phosphorylated CaMKKβ and AMPKα, and increased the level of phosphorylated mTOR and p70S6K under ADM exposure (Fig. 4D). Addition of the intracellular Ca^2+^ chelator BAPTA/AM markedly inhibited the activity of CaMKKβ/AMPKα/mTOR pathway and reduced the LC3-II levels (Fig. 4E). Thus, [Ca^2+^]_i_ regulated by TRPC5 may be most likely required for the initiation of autophagy in response to chemotherapy. When we silenced CaMKKβ using siRNA in MCF-7 and MDA-MB 231 cells, the activity of AMPKα and the accumulation of LC3-II were inhibited and the activity of mTOR was enhanced on exposure to ADM (Fig. 4F). Similar to the effect of CaMKKβ-silencing, knockdown of AMPKα also significantly augmented the phosphorylated mTOR levels and attenuated the accumulation of LC3-II compared with the siRNA control under ADM exposure (Fig. 4G). Moreover, the number of LC3 dots per cell in response to ADM was significantly decreased after knockdown of CaMKKβ or AMPKα (Fig. 4H and I). Addition of BAPTA/AM also reduced the number of LC3 dots per cell with exposure to ADM in breast cancer cells (Fig. 4H and I). STO-609, an inhibitor of CaMKKβ, and compound C, an inhibitor of AMPK also attenuated the numbers of LC3 puncta in MCF-7 and MDA-MB 231 cells (Fig. 4J and K). Moreover, BAPTA/AM, STO-609, and compound C also decreased the level of LC3-II in drug-resistant MCF-7/ADM cells (Fig. 4L). In addition, we found that BAPTA/AM and knockdown of CaMKKβ or AMPKα reduced viability of MCF-7 and MDA-MB 231 cells with ADM exposure (Fig. 5A to C). Clonogenicity also decreased in these cells (Fig. 5E,G and I). Notably, inhibition of the CaMKKβ/AMPKα/mTOR pathway also enhanced the sensitivity of drug-resistant MCF-7/ADM cells to ADM (Fig. 5D,F and H). These results revealed that TRPC5 mediates the cytoprotective autophagy during ADM exposure *via* the CaMKKβ/AMPKα/mTOR pathway.

### Suppression of autophagy by down-regulated TRPC5 increases sensitivity to ADM *in vivo*

**Figure 6:**
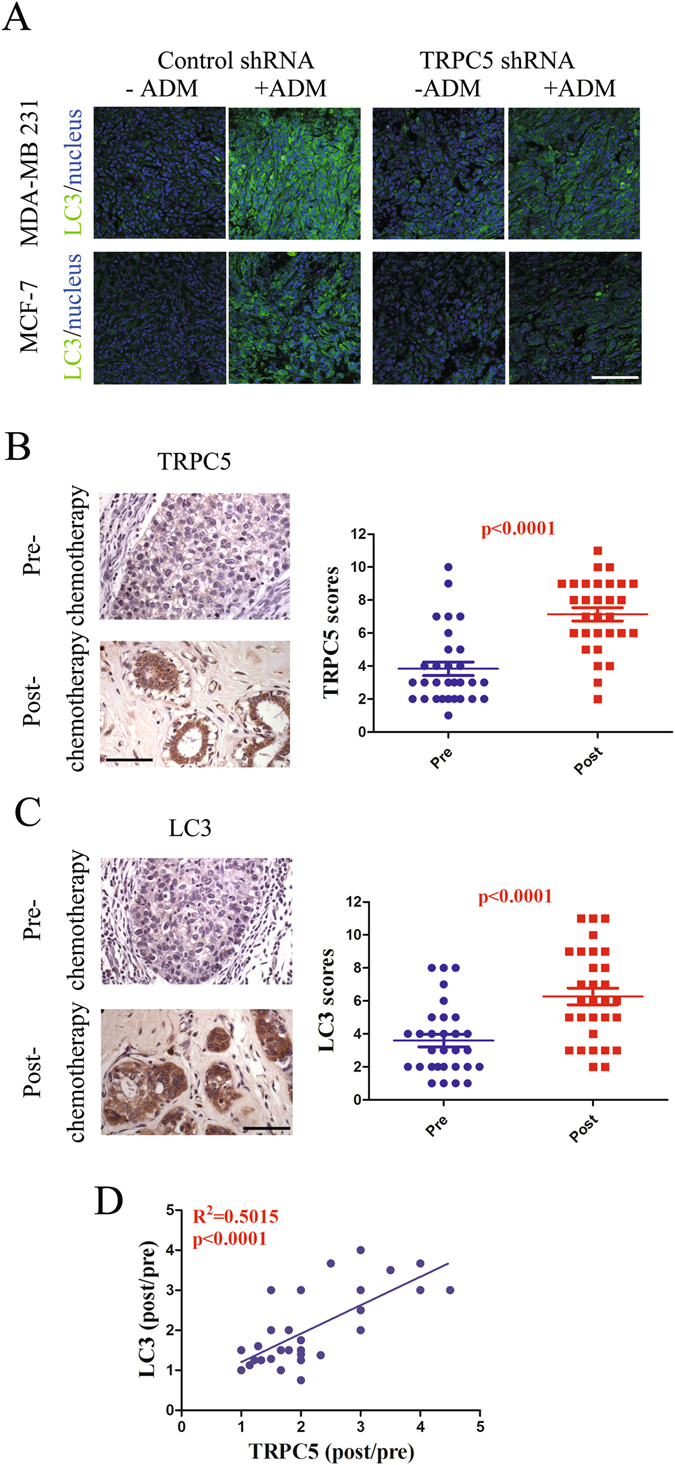
Suppression of autophagy by down-regulated TRPC5 increases sensitivity to ADM *in vivo*. (**A**) Female nude mice were inoculated with MCF-7 or MDA-MB 231 cells transfected with control or TRPC5 shRNA lentiviral particles and treated with ADM (6 mg/kg) when the tumors reached ~100 mm^3^ (n = 5 in each group). Autophagy in tumor samples were assayed by LC3 stain. Scale bar: 100 μm. (**B** and **C**) Representative images and summary data from immunohistochemical staining of TRPC5 and LC3 in paired pre- and post-chemotherapy breast cancer tissue from patients showing elevated TRPC5 or LC3 expression (n = 31). Scale bar: 100 μm. (**D**) Pearson correction of TRPC5 expression with LC3 (n = 31). Data were analyzed using Pearson correlation test. Values are mean ± SEM *p < 0.05, compared to control shRNA, ^#^P < 0.05, compared to TRPC5 shRNA.

To explore whether the targeted inhibition of TRPC5-induced autophagy also enhances sensitivity to ADM *in vivo*, we subcutaneously injected nude mice with MCF-7 and MDA-MB 231 cells that had previously been transfected with TRPC5 shRNA lentiviral particles. The growth of TRPC5-knockdown cancer cells after ADM exposure was markedly less than in cells transfected with control shRNA (Supplemental Figure S9). We found that cancer cells transfected with TRPC5 shRNA showed attenuated autophagy with ADM exposure (Fig. 6A). Notably, the expression of LC3 was significantly higher in drug-resistant MCF-7/ADM xenografts than in MCF-7 xenografts (Supplemental Figure S10). In order to investigate the clinical potential of TRPC5 in the induction of autophagy in breast cancer, we analyzed breast cancer tissue from 31 paired patients with or without anthracycline-taxane-based chemotherapy (Supplementary Table S1). The results showed that both TRPC5 and LC3 expression was markedly up-regulated after chemotherapy (Fig. 6B and C). In addition, we found statistically significant positive correlations between TRPC5 and LC3 expression (Fig. 6D).

## Discussion

Many breast cancer patients acquire resistance to chemotherapeutic drugs and this phenomenon is the major cause of death. Although chemotherapy-induced autophagy is now recognized to be an important contributor to chemotherapy resistance in cancer cells, the underlying mechanism and the inducer of this effect remain unclear. Here, we showed that TRPC5-regulated autophagy is an important contributor to the development and maintenance of drug resistance in breast cancer. Blockade of TRPC5 and autophagy augmented breast cancer cell death in response to chemotherapy. We also found significant positive correlations between TRPC5 and autophagy in patients before and after neoadjuvant chemotherapy.

Different mechanisms involving dysfunctional drug transports, resistance to cell death, and the production of cancer stem like cells have been reported to contribute to chemoresistance. Our previous studies showed that the overexpression of TRPC5 up-regulates the level of p-glycoprotein to maintain chemoresistance in drug-resistant MCF-7/ADM cells, suggesting that TRPC5 plays a role in regulating drug transporters. Greka A *et al*. reported that TRPC5 regulates neurite outgrowth^39^. Here, we found that ADM exposure enhanced the expression of TRPC5 in drug sensitive breast cancer cells (MCF-7, T47D and MDA-MB 231 cells). Moreover, silencing of TRPC5 expression enhanced death and suppressed recovery in breast cancer cells under chemotherapy. Similar results were found in drug-resistant MCF-7/ADM cells. Therefore, we demonstrated that TRPC5 is a negative regulator of drug-induced cell death in breast cancer cells. Cell death is one of the mechanisms by which chemotherapy destroys cancer cells. Augmented autophagy occurs in response to chemotherapy in breast cancer cells. It is believed that autophagy blocks the accumulation of redundant or damaged components and facilitates the recycling of these components to sustain survival^18–21^. However, chemotherapeutic agents can decrease the autophagy in breast cancer cells^40^. In line with the reports of up-regulated autophagy with exposure to chemotherapy, we found that ADM exposure increased LC3-II expression and LC3 puncta formation in breast cancer cells^32, 33^. Drug-resistant MCF-7/ADM cells maintained a higher level of autophagy than MCF-7 cells. Combined addition of CQ or 3-MA with ADM decreased the recovery and viability of both sensitive or resistant breast cancer cells compared with ADM alone, confirming that autophagy is mainly a cytoprotective process^41^. Next, we explored the relationship between TRPC5 and autophagy. We found that knockdown of TRPC5 expression reduced LC3-II levels and LC3 puncta formation in response to ADM. Overexpression of TRPC5 significantly increased LC3-II levels and LC3 puncta formation and facilitated futher resistance of breast cancer cells to ADM. In addition, we found that cancer cells transfected with TRPC5 shRNA lentiviral particles showed attenuated autophagy and tumor size with ADM exposure. Moreover, we found significant positive correlations between TRPC5 and autophagy in patients before and after neoadjuvant chemotherapy. Therefore, TRPC5 potentiates sensitivity to ADM *via* the regulation of autophagy in breast cancer cells.

Autophagosome formation is negatively regulated by mTOR, which directly regulates ULK1-ATG13-FIP200 complex^42^. However, reports have also shown that the inhibition by mTOR is not involved in the autophagy induced by lipopolysaccharide and EEF2K (eukaryotic elongation factor-2 kinase) silencing^29^. Therefore, the function of mTOR in autophagy may depend on the cell type and setting. AMPK functions as an antagonist of mTOR, and is a key player in the stimulation of autophagy^43, 44^. AMPKα is phosphorylated and activated by CaMKKβ in response to increased [Ca^2+^]_i_^45^. The regulation of Ca^2+^ homeostasis by TRPC5 in response to many physiological stimuli has been confirmed^46^. Our previous data have also shown that drug-resistant cancer cells produce abundant p-glycoprotein *via* TRPC5-related Ca^2+^ signaling^15^. Moreover, Ca^2+^-mobilizing agents induce massive accumulation of autophagosomes in a Beclin 1- and Atg7 -dependent manner^47^. In addition, CaMKKβ occurs downstream from TRPC5^34^. Therefore, to test whether the induction of autophagy by TRPC5 under chemotherapy was depends on the CaMKKβ/AMPKα/mTOR pathway, we examined the effects of pharmacological inhibition and gene silencing on this pathway. Basal Ca^2+^ was significantly elevated after ADM exposure, and this was primarily regulated by TRPC5. This finding suggested that TRPC5 is mainly responsible for the increase in [Ca^2+^]_i_. However, knockdown of TRPC5 did not completely abolish the increase in [Ca^2+^]_i_, suggesting TRPC5 play a major role in ADM-induced [Ca^2+^]_i_ raise. Other TRP or store operated calcium channels may be partly involved in this process. Under hypoxia and nutrient depletion, TRPC1 regulates autophagy to protect against cell death^48^. TRPC4 is involved in regulation of autophagy by Trans-3,5,4′-trimethoxystilbene in endothelial cells^49^. Future study is needed to further clarify this issue. Knockdown of TRPC5 suppressed the activity of CaMKKβ and AMPKα, increased activity of mTOR with ADM exposure. Furthermore, BAPTA/AM, STO-609, or silencing CaMKKβ, and compound C, or silencing AMPKα, inhibited the initiation of autophagy and enhanced cell death in response to ADM. In agreement with the previous findings that inhibition of mTOR induces autophagy, our data showed that the autophagy induced by TRPC5 is dependent on mTOR inhibition in breast cancer cells undergoing chemotherapy. Here, our findings strongly support the idea that the CaMKKβ/AMPKα/mTOR pathway is required for TRPC5-induced autophagy in response to chemotherapy.

**Figure 7:**
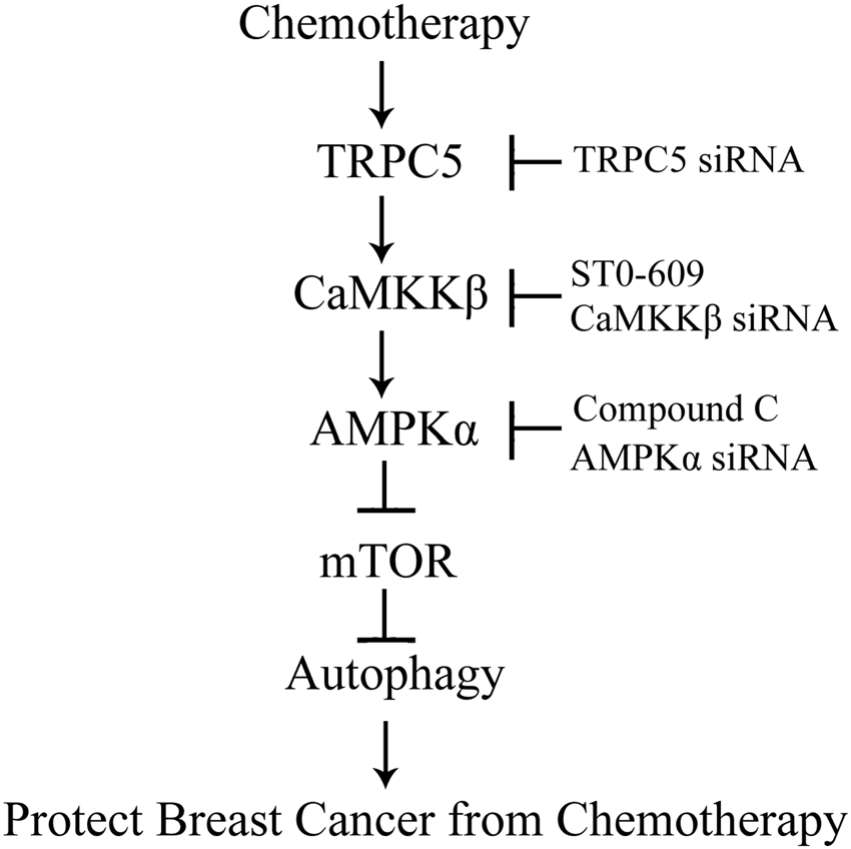
Signaling connections involved in TRPC5-initiated autophagy pathways in response to chemotherapy in breast cancer cells. Chemotherapy up-regulates TRPC5 expression and then increases the basal [Ca^2+^]_i_. The increased [Ca^2+^]_i_ actives CaMKKβ by phosphorylation, which in turn activates AMPKα by phosphorylation. Activation of AMPKα negatively regulates mTOR by suppressing its phosphorylation, leading to autophagy. Autophagy induced by TRPC5 during chemotherapy promotes the survival of human breast cancer cell. Arrows represent promotion events, blunt arrows indicate suppression events.

In conclusion, our findings showed that TRPC5-induced autophagy counteracts the antiproliferative effects of ADM *via* the CaMKKβ/AMPKα/mTOR pathway in breast cancer cells (Fig. 7). TRPC5 is positively correlated with autophagy in patients before and after neoadjuvant chemotherapy. Our current findings reveal a novel role of TRPC5 as an inducer of autophagy, and suggest a novel mechanism of drug resistance in chemotherapy for breast cancer.

## Materials and Methods

### Ethics statement

The animal experiments conformed to the Guide for Animal Care and Use of Laboratory Animals published by the National Institutes of Health, USA. All procedures were approved by the Animal Experimentation Ethics Committee of The Chinese University of Hong Kong. The study using clinical samples was approved by the Review Board of the Affiliated Hospital of Jiangnan University.

### Patients

The human breast tumor samples (n = 31) were from Affiliated Hospital of Jiangnan University. Informed consent was requested as anonymous specimens and was given by all human participants in this study. Patients were recruited between 2010 and 2013. The protocol for all patients included 1–6 cycles of anthracycline-taxane-based chemotherapy. Tumors were assessed was by MRI and/or ultrasound depending on that used at baseline. Treatment response was assessed using the RECIST criteria^50^.

### Reagents and antibodies

Alexa Fluor 488 donkey anti-rabbit IgG (A21206) antibody was from Life Technologies Corp; fetal bovine serum (16000-044), Dulbecco’s Modified Eagle medium (DMEM; 11995-040) and RPMI 1640 medium (11875-093) from Gibco. 3-(4,5-dimethylthiazol-2-yl)-2,5-diphenyl-2H-tetrazolium bromide (MTT; M2128), pepstatin A (P5318), 3-Methyladenine (3-MA; M9281) and anti-LC3B antibody (L7543) from Sigma; BAPTA/AM (196419) and compound C (171260) from Calbiochem; E64d (4545) and STO-609 (1551) form Tocris Bioscience; anti-TRPC5 (ACC-020) form Alomone labs; anti-ATCB (sc-47778) from Santa Cruz Biotechnology; anti-AMPKα (#2532 S), anti-phospho-AMPKα (Thr172) (#2535), anti-mTOR (#2972), anti-phospho-mTOR (Ser2448) (#2971), anti-CaMKKβ (#4436), anti-phospho-CaMKKβ (Thr286) (#12716), anti-phospho-p70S6K (Thr389) (#9205), anti-p70S6K (#9202) from Cell Signaling Technology.

### Cell culture

MCF-7, T47D and MDA-MB 231 cells were from the American Type Culture Collection, ADM-resistant human breast cancer cells (MCF-7/ADM cells) were derived by treating MCF-7 cells with step wise increasing concentrations of ADM over 8 months. MCF-7, T47D, and MCF-7/ADM cells were cultured in RPMI 1640 supplemented with 10% FBS, 100 μg/ml penicillin and 100 U/ml streptomycin. MDA-MB 231 cells were cultured in DMEM supplemented with 10% FBS, 100 μg/ml penicillin and 100 U/ml streptomycin.

### Cell viability (MTT) assay

Cells were seeded in 96-well plates at 5000 cells per well and were treated as indicated for 24 to 72 h depending on the experimental conditions. MTT (20 µL, 5 mg/mL) was added to each well and incubated for 4 h. Finally, the medium from each well was replaced by 150 µL DMSO to dissolve the formazan before measurement on a microplate reader (Bio-Rad Laboratories) at 490 nm. The cell viability was normalized to the control group.

### Small-interfering RNA (siRNA) transfection

Cells were transiently transfected with gene-specific or scrambled siRNA using DharmaFECT 1 Transfection Reagent (GE Healthcare) following the procedure recommended by the manufacturer. TRPC5 silencing was performed using siRNA duplexes targeting the following sequences: 5′-CCAAUGGACUGAACCAGCUUUACUU-3′ and 5′-UGUCGUGGAA UGGAUGAUAUU-3′. CaMKKβ silencing was performed using siRNA duplexes targeting the following sequences: 5′-CGAUCGUCAUCUCUGGUUA-3′ and 5′-GGAUCUGAUCAAAGGCAUC-3′. AMPKα silencing was performed using siRNA duplexes targeting the following sequences: 5′-GAGGAGAGCUAUUUGAUUA -3′ and 5′-GCUGUUUGGUGUAGGUAAAC-3′. In brief, cells were transfected in RPMI 1640 or DMEM with 100 nM of each siRNA duplex using DharmaFECT transfection reagent according to the manufacturer’s protocol.

### Overexpression of human TRPC5

A plasmid pcDNA3.1-TRPC5 containing the full-length human TRPC5 coding region (NM_012471.2) was from GenScript Co. Transfection with pcDNA3.1-TRPC5 plasmid was carried out using the Lipofectamine 2000 Transfection Reagent (Invitrogen) according the manufacturer’s instructions.

### Immunohistochemical Staining

Tissue slides were deparaffinized with xylene and rehydrated through a graded alcohol series. The endogenous peroxidase activity was blocked by incubation in 3% (vol/vol) hydrogen peroxide for 10 min. Antigen retrieval was carried out by immersing the slides in 10 mM sodium citrate buffer (pH 6.0) and maintaining them at a sub-boiling temperature for 10 min. The slides were incubated with the primary antibody in 5% (wt/vol) BSA and 0.4% sodium azide in PBS at 4 °C in a humidified chamber. Subsequently, the sections were incubated with the GTVision III Detection System/Mo&Rb Kit (Gene Tech Co., Ltd). All staining was assessed by pathologists blinded to the origin of the samples and patient outcomes. The widely-accepted German semi-quantitative scoring system was used to assess the staining intensity and proportion of stained cells. Each specimen was assigned a score according to the intensity of staining (0, none; 1, weak; 2, moderate; 3, strong) and the proportion of stained cells (0, 0%; 1, 1–24%; 2, 25–49%; 3, 50–74%; 4, 75–100%). The final score for immunoreactivity was determined by multiplying the intensity by the proportion, ranging from 0 to 12^17^.

### Immunofluorescence analysis

Briefly, cultured cells or frozen sections of xenografts were fixed in 4% paraformaldehyde (PFA; Sigma-Aldrich) for 15 min then blocked in 5% BSA with 0.1% Triton X-100 (Bio-Rad) in PBS for 30 min at room temperature. Samples were incubated with primary antibodies overnight at 4 °C followed by the appropriate secondary fluorescently-labeled antibody (Invitrogen Molecular Probes) for 1 h at room temperature. Nuclei were counterstained with DAPI. Images were captured on an Olympus FV1000 confocal microscope for cultured cells and a Leica TCS SP8 confocal microscope for frozen sections. LC3 dots were analyzed in a blinded manner by manual counting and the average number of LC3 dots per cell was counted in more than 5 fields with at least 90 cells for each group.

### Western blot analysis

Cells were lysed in a detergent extraction buffer containing 1% (vol/vol) Nonidet P-40, 150 mmol/L NaCl, and 20 mmol/L Tris–HCl, pH 8.0, with protease inhibitor cocktail tablets for 30 min on ice and centrifuged for 15 min at 4 °C. Protein concentrations were then measured using a Bio-Rad protein assay kit (Hercules, CA). Proteins were separated on an 8–12% gel using sodium dodecyl sulfate polyacrylamide gel electrophoresis. For immunoblots, the polyvinylidene difluoride membrane carrying the transferred proteins was incubated at 4 °C overnight with designated primary antibodies diluted in TBST buffer pH 7.5, containing 50 mM Tris, 150 mM NaCl, 0.1% Tween20, and 5% BSA. Immunodetection was accomplished using a horseradish peroxidase-conjugated secondary antibody and an enhanced chemiluminescence detection system (GE Healthcare)^51^. Densitometry analyses were performed using ImageJ software (NIH), and ACTB control was used to confirm equal sample loading and normalization of the data.

### Clonogenic and crystal violet proliferation recovery assay

Cells were transfected with siRNAs or plasmid for 48 h, and then seeded at appropriate dilutions onto 6-well plates. After 24 h, ADM was added and the cells were incubated for 4 days. If needed, cells were treated with 20 µmol/L BAPTA/AM for 2 h before adding ADM. Medium with ADM was replaced with fresh medium without ADM and cultured for another 7 to 9 days. Colonies were fixed with glutaraldehyde (6.0% v/v), stained with crystal violet (0.5% w/v) and imaged. Colonies with 50 or more cells were counted.

### Analysis of mRNA expression by real-time PCR

To determine the mRNA expression of TRPC5, real-time PCR analysis was performed. Total RNA was isolated from cells using TRIzol Reagent (Invitrogen), and treated with DNase I (Invitrogen). cDNA was synthesized from 1 µg total RNA, using random primers with a High Capacity cDNA Reverse Transcription Kit (Applied Biosystems). Gene expression was normalized against ACTB. The primer sequences used were: ACTB forward 5′-CACCATTGGCAATGAGCGGTTC-3′, reverse 5′-AGGTCTTTGCGGATGTCCACGT-3′; TRPC5 forward 5′-TGAACTCCCTCTACCTGGCAAC-3′; reverse 5′-CGAAGAGTGCTTCCGCAATCAGT-3′; LC3 forward 5′-TACGAGCAGGAGAAAGACGAGG-3′; reverse 5′-GGCAGAGTARGGTGGGTTGGTG-3′. Real-time PCR was performed with 7500 Fast Real-time PCR system (Applied Biosystems), using Power SYBR Green PCR Master Mix (Applied Biosystems).

### [Ca^2+^]_i_ measurement

[Ca^2+^]_i_ in cultured cells was measured as described elsewhere^52^. Briefly, MCF-7 or MDA-MB 231 cells were loaded with 10 µM Fura-2/AM and 0.02% pluronic F-127 for 1 hour in dark at 37 °C in NPSS. Fura-2 fluorescence signals were measured using dual excitation wavelengths at 340 and 380 nm using an Olympus fluorescence imaging system. 10 to 20 cells were analyzed in each experiment.

### Mouse xenograft models

To generate subcutaneous tumors, MCF-7 or MDA-MB 231 cells were first transfected with control or TRPC5 shRNA Lentiviral Particles (sc-42670-v, Santa Cruz) for 48 h, then 5 × 10^6^ these cells were injected into the flank of female nude mice with or without estrogen supplementation. All mice were housed in air-filtered pathogen-free condition. Tumor growth was monitored with digital calipers every 5 days. Tumor volumes were estimated using the formula: volume (mm^3^) = (width)^2^ × length/2 and tumor growth was plotted against time. When the tumors reached ~100 mm^3^, the mice with tumors derived from MCF-7 or MDA-MB 231 cells were injected with 6 mg/kg ADM (i.p., once every 3 days); nude mice bearing xenograft tumors derived from MCF-7/ADM cells were injected with 3 mg/kg ADM (i.p., once every 3 days)^15, 17^.

### Statistical Analyses

We determined the correlations between TRPC5 expression and LC3 expression using the Pearson correlation test. Statistical analysis was performed using the 2-tailed Student’s t-test or one-way ANOVA. All analyses were performed using GraphPad Prism version 5. Results are presented as mean ± SEM of at least 3 independent experiments. All tests were two-sided, and P values < 0.05 were considered to be statistically significant.

## Electronic supplementary material

supplementary data
