# Supplementary material for: TRPC5-induced autophagy promotes drug resistance in breast carcinoma via CaMKKβ/AMPKα/mTOR pathway
Source: Sci Rep. 2017 Jun 9;7:3158. doi: 10.1038/s41598-017-03230-w (PMC5466655; doi:10.1038/s41598-017-03230-w)
Supplement: Supplementary file 1 — supplementary data [file 41598_2017_3230_MOESM1_ESM.doc]

**TRPC5-induced autophagy promotes drug resistance in breast**

**carcinoma *via* CaMKKβ/AMPKα/mTOR pathway**

Peng Zhang^1†^, Xiaoyu Liu^3†^, Hongjuan Li^2^, Zhen Chen^2^, Xiaoqiang Yao^3^, Jian Jin^2*^, Xin Ma^1*^

^1^Wuxi Medical School, Jiangnan University, Wuxi, China

^2^School of Pharmaceutical Sciences, Jiangnan University, Wuxi, China

^3^School of Biomedical Sciences, The Chinese University of Hong Kong, Shatin, New Territories, Hong Kong, China

^†^These authors contributed equally to this work

^*^To whom correspondence should be addressed: XM: Wuxi Medical School, Jiangnan University, No. 1800 Lihu Avenue, Wuxi, China. Tel.: 86-510-85918219; Fax: 86-510-85918219; E-mail: [maxin@jiangnan.edu.cn](mailto:maxin@jiangnan.edu.cn); JJ: School of Pharmaceutical Sciences, Jiangnan University, No. 1800 Lihu Avenue, Wuxi, China. Tel.: 86-510-85918219; Fax: 86-510-85918219; E-mail: [jinjian31@126.com](mailto:maxin@jiangnan.edu.cn);

Running title: TRPC5-induced autophagy promotes drug resistance

**Supplementary data**


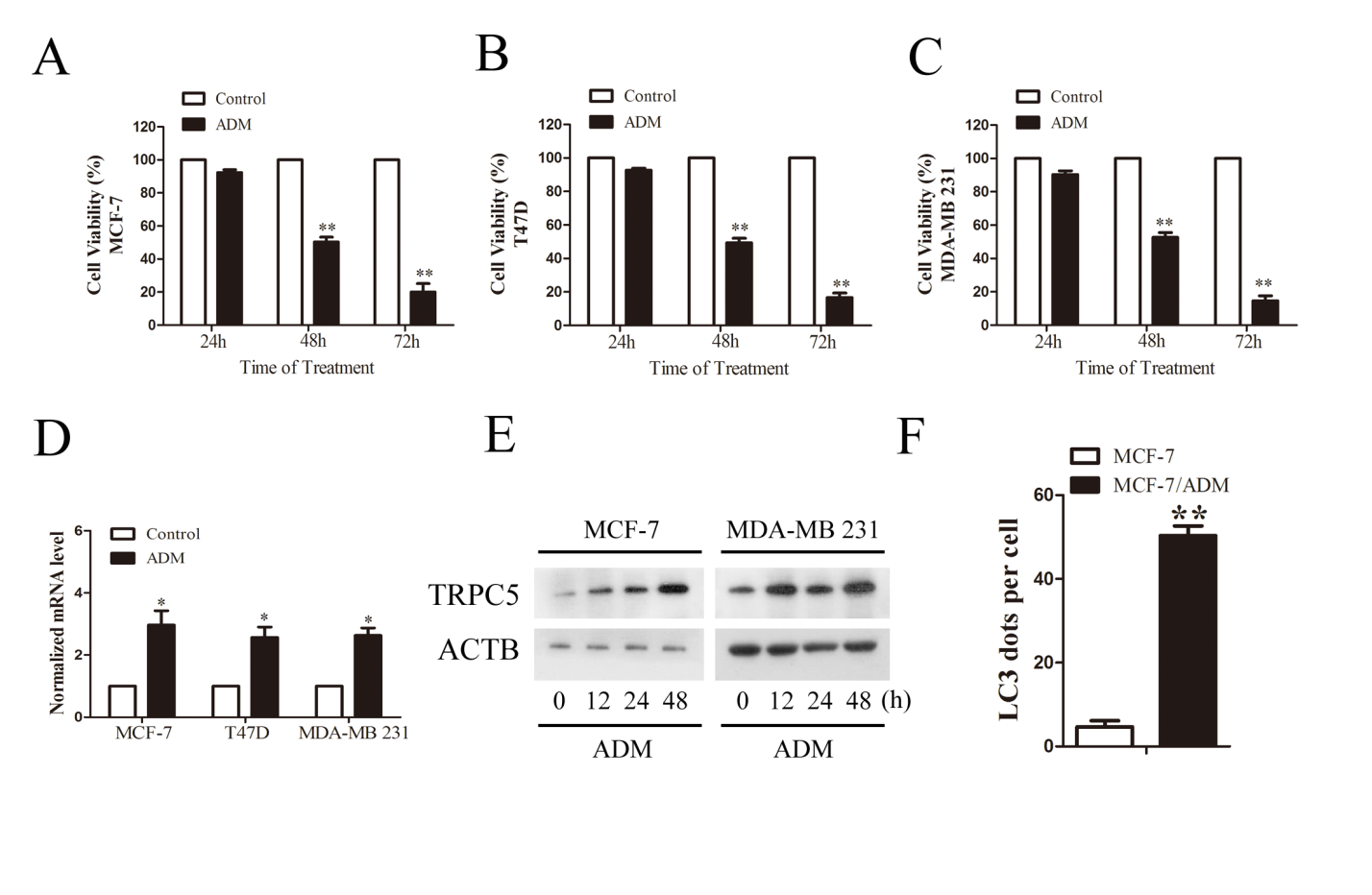


Figure S1 MTT assay. MCF-7, T47D and MDA-MB 231 cells were treated with 400 , 300 and 800 nmol/L Adriamycin (ADM) respectively, for 24h, 48h and 72h.Values are means ± SEM of 4 experiments **, p<0.01, compared to control


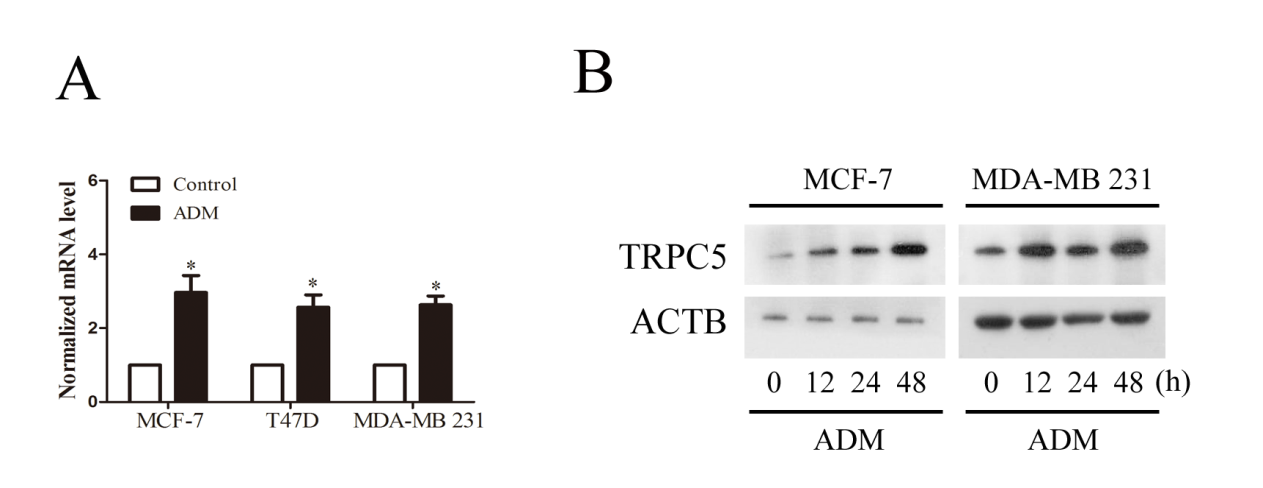


Figure S2 (A) Breast cancer cells treated with ADM as in figure S1 for 48h and then TRPC5 mRNA was analyzed by real-time PCR. (B)MCF-7 and MDA-MB 231 cells were treated with ADM for 12 to 48h and then TRPC5 protein was analyzed by western blotting. Values are means ± SEM of 3 to 5 experiments. *, p<0.05, compared to control


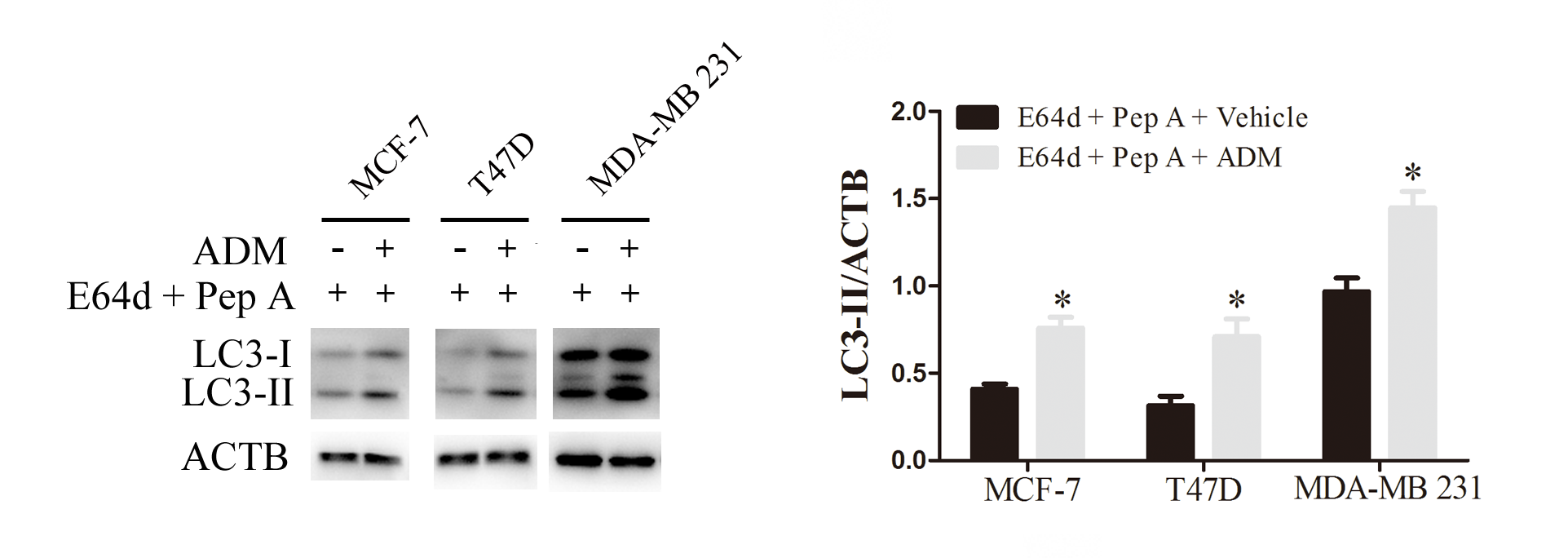


Figure S3 Representative western blots and densitometric analysis normalized to ACTB demonstrating the effect of the lysosomal protease inhibitors 10 μg/mL E64d plus pepstatinA (Pep A) on ADM-induced LC3-II accumulation. Values are means ± SEM of 4 experiments. *, p<0.05, compared to Vehicle.


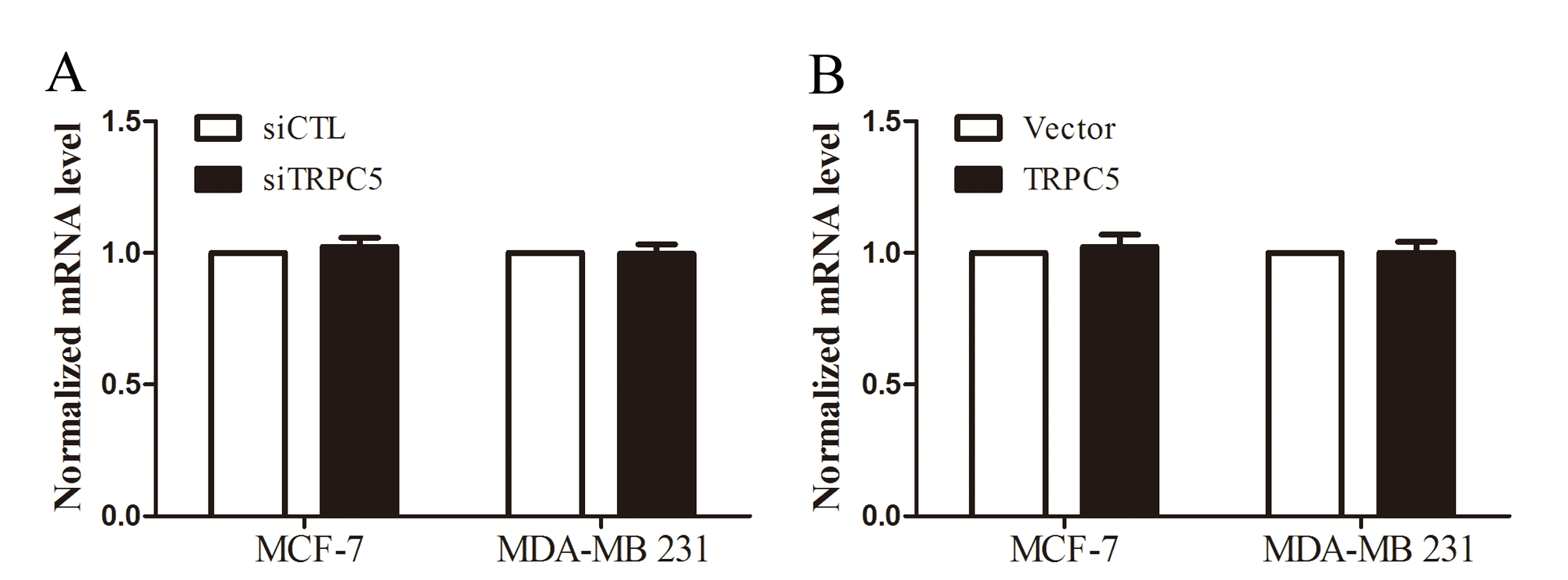


Figure S4 (A) MCF-7 and MDA-MB 231 cells were transfected with siTRPC5 or siCTL for 48h and then LC3 mRNA was analyzed by real-time PCR. (B) MCF-7 and MDA-MB 231 cells were transfected with TRPC5-plasmid or vector-plasmid for 48h and then LC3 mRNA was analyzed by real-time PCR. Values are means ± SEM of 4 experiments.


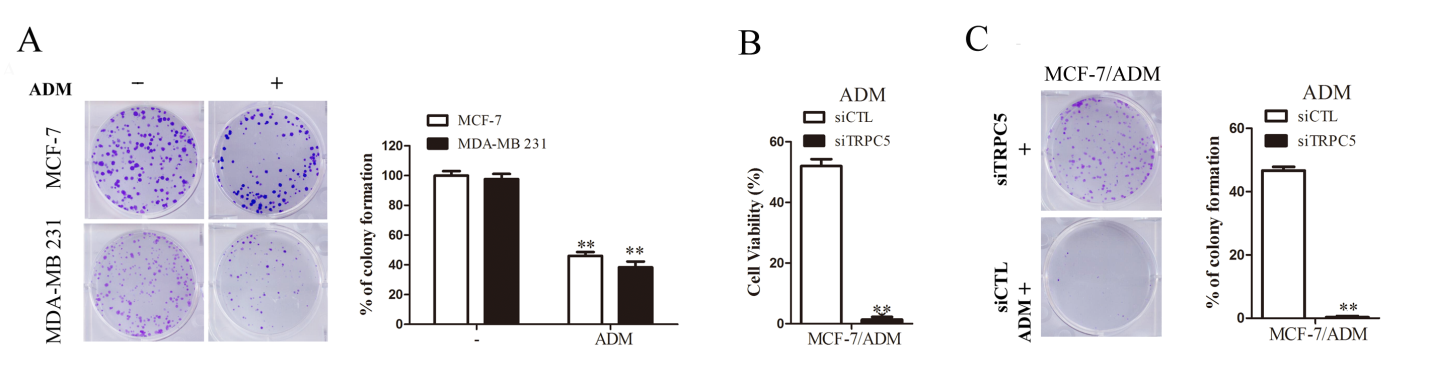


Figure S5 (A) clonogenic recovery assay. MCF-7 and MDA-MB 231 cells were treated with 5 nmol/L and 10 nmol/L ADM respectively. (B) MTT assay. MCF-7/ADM cells were transfected with siTRPC5 or siCTL for 24 h and then treated with 300µmol/L ADM respectively for 48 h. (C) clonogenic recovery assay. MCF-7/ADM cells transfected with siTRPC5 or siCTL for 24 h and then treated with 10 µmol/L ADM. Values are means ± SEM of 3 to 6 experiments. **, p<0.01, compared to control or siCTL.


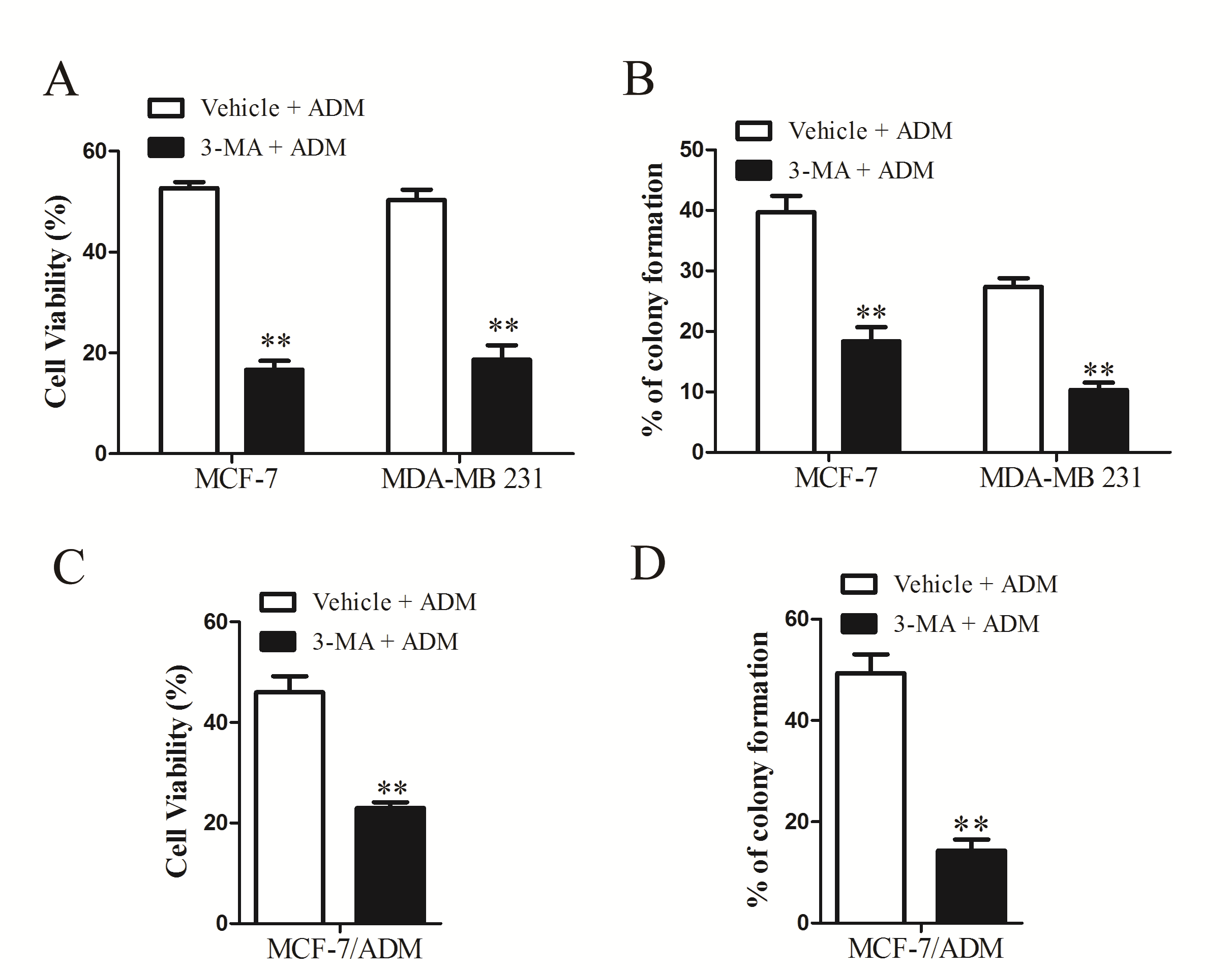


Figure S6 (A) The effect of 1 mmol/L 3-MA on cell viability. (B) The effect of 50 µmol/L 3-MA on clonogenic recovery in indicated cells. (C and D) The effect of 1 mmol/L 3-MA on viability and 50 µmol/L 3-MA on clonogenic recovery in MCF-7/ADM cells. Values are mean ± SEM of 4 experiments. **,p<0.01, compared to Vehicle.


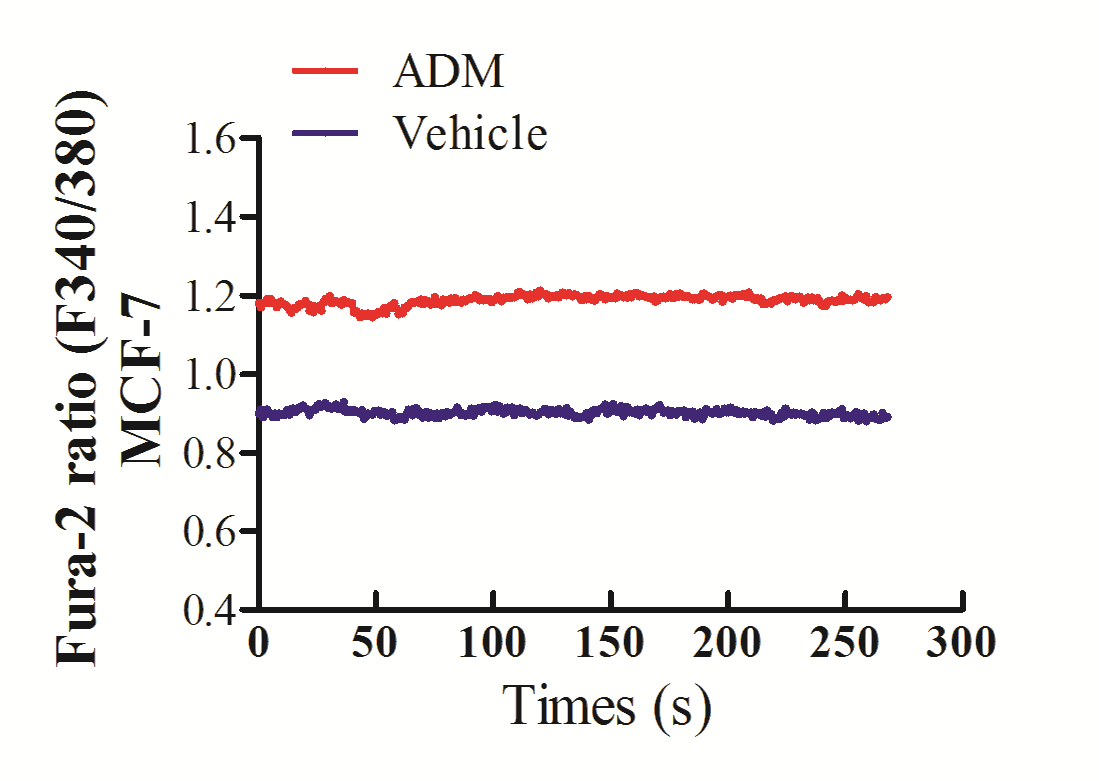


Figure S7 Representative traces illustrating the basal [Ca^2+^]_i_ from vehicle or ADM treated for 48h in MCF-7 cells.


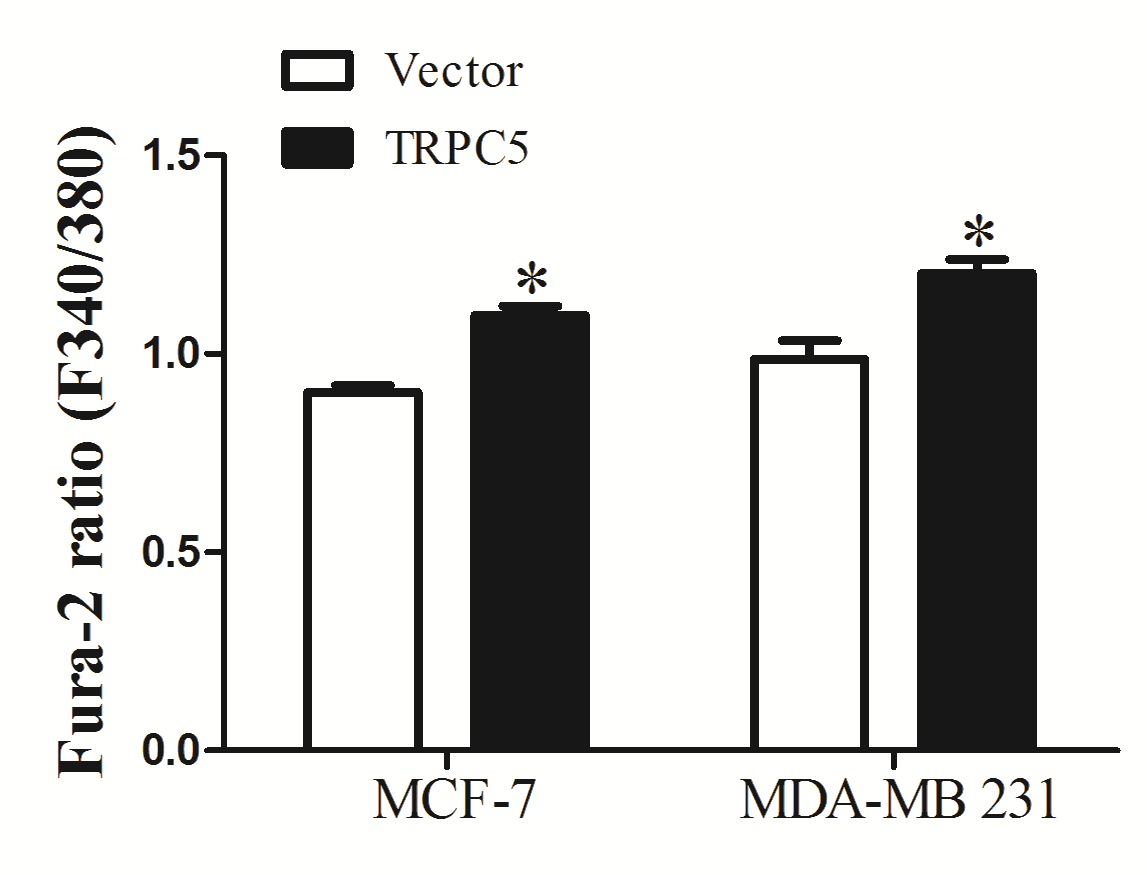


Figure S8 The effect of TRPC5 overexpression on the basal [Ca^2+^]_i_ in MCF-7 and MDA-MB 231 cells. Values are means ± SEM of 4 experiments. *, p<0.05, compared to Vector.


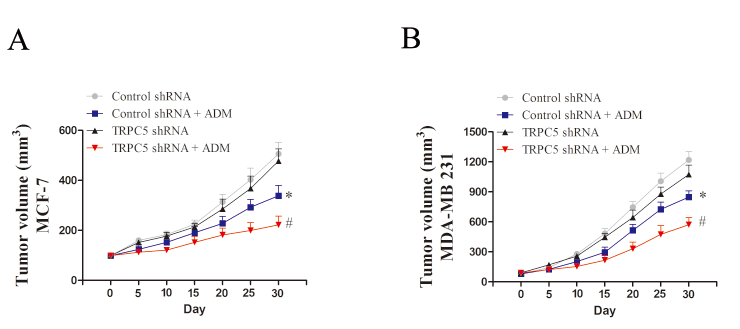


Figure S9 (A and B) Profiles of tumor volumes. Female nude mice were inoculated with MCF-7 or MDA-MB 231 cells transfected with control or TRPC5 shRNA and then treated with ADM (6mg/kg) when the tumors reached ∼100 mm^3^(n=5 in each group). Values are mean ± SEM; *p<0.05 compared to Control shRNA , #p<0.05 compared to Control shRNA+ADM


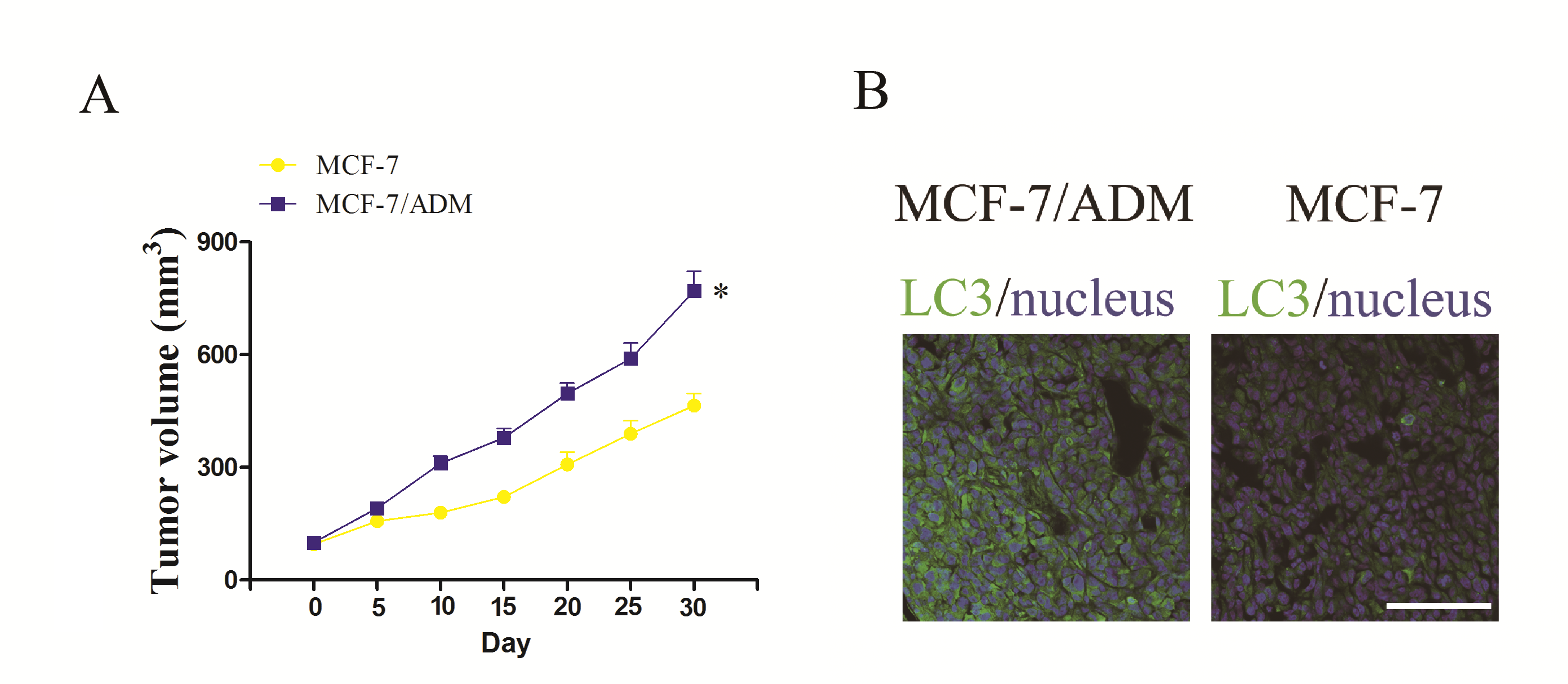


Figure S10 (A) Profiles of tumor volumes. Female nude mice were inoculated with MCF-7 or MCF-7/ADM cells (n=5 in each group). Xenograft tumors derived from MCF-7/ADM cells were injected with 3mg/kg ADM (i.p., once every 3 days. (B) Autophagy was assayed by LC3 stain in the MCF-7 and MCF-7/ADM xenografts. Scale bar: 100 μm. Values are mean ± SEM; *p<0.05 compared to MCF-7

**Supplementary Table 1** Clinical, pathological and biomarker characteristics of 31 patients with paired breast cancer tissue pre- and post-neoadjuvant chemotherapy at the time of primary diagnosis of breast cancer.

Tumor characteristics for breast cancer patients

|  | All Patients | | Resistance Patients | | Sensitive Patients | |
| --- | --- | --- | --- | --- | --- | --- |
|  | (n=31) | | (n=18) | | (n=13) | |
| Age(years) |  | |  | |  | |
| <50 | 13(41.9%) | | 5(27.8%) | | 9(69.2%) | |
| ≥50 | 18(58.1%) | | 13(72.2%) | | 4(31.8%) | |
| Sex |  | |  | |  | |
| Male | 0(0.0%) | | 0(0.0%) | | 0(0.0%) | |
| Female | 31(100.0%) | | 18(100.0%) | | 13(100.0%) | |
| Histology |  | |  | |  | |
| Ductal | 31(100.0%) | | 18(100.0%) | | 13(100.0%) | |
| Breast cancer | Pre | Post | Pre | Post | Pre | Post |
| Tumour size |  |  |  |  |  |  |
| T1 | 4(12.9%) | 5(16.1%) | 2(11.1%) | 2(11.1%) | 2(15.3%) | 3(23.1%) |
| T2 | 8(25.8%) | 9(29.0%) | 5(27.8%) | 5(27.8%) | 3(23.1%) | 4(30.8%) |
| T3 | 12(38.7%) | 12(38.7%) | 7(38.9%) | 8(44.4%) | 5(38.5%) | 4(30.8%) |
| T4 | 7(22.6%) | 5(16.2%) | 4(22.2%) | 3(16.7%) | 3(23.1%) | 2(15.3%) |
| Lymph node status |  |  |  |  |  |  |
| N0 | 7(22.6%) | 3(9.8%) | 5(27.8%) | 0(0.0%) | 2(15.3%) | 3(23.1%) |
| N1 | 4(12.9%) | 7(22.6%) | 2(11.1%) | 4(22.2%) | 2(15.3%) | 3(23.1%) |
| N2 | 14(45.2%) | 13(41.9%) | 8(44.4%) | 8(44.4%) | 6(46.3%) | 5(38.5%) |
| N3 | 6(19.3%) | 8(25.7%) | 3(16.7%) | 6(33.4%) | 3(23.1%) | 2(15.3%) |
| AJCC Substage |  |  |  |  |  |  |
| I | 0(0.0%) | 1(3.2 %) | 0(0.0%) | 0(0.0%) | 0(0.0%) | 1(7.7%) |
| II | 4(12.9%) | 5(16.1%) | 3(16.7%) | 1(5.6%) | 1(7.7%) | 4(30.8%) |
| III | 27(87.1%) | 25(80.7%) | 15(83.3%) | 17(94.4%) | 12(92.3%) | 8(61.5%) |
